# Expression, Processing, and Localization of PmpD of *Chlamydia trachomatis* Serovar L2 during the Chlamydial Developmental Cycle

**DOI:** 10.1371/journal.pone.0000568

**Published:** 2007-06-27

**Authors:** Andrey O. Kiselev, Walter E. Stamm, John R. Yates, Mary F. Lampe

**Affiliations:** 1 Department of Laboratory Medicine, University of Washington, Seattle, Washington, United States of America; 2 Division of Allergy & Infectious Diseases, Department of Medicine, University of Washington, Seattle, Washington, United States of America; 3 Department of Cell Biology, The Scripps Research Institute, La Jolla, California, United States of America; Tufts University, United States of America

## Abstract

**Background:**

While families of polymorphic membrane protein (*pmp*) genes have been identified in several *Chlamydia* species, their function remains mostly unknown. These proteins are of great interest, however, because of their location in the outer membrane and possible role in chlamydial virulence.

**Methodology/Principal Finding:**

We analyzed the relative transcription of the *pmpD* gene, a member of the *pmp* gene family in *C. trachomatis* serovar L2, and its protein product translation and processing during the chlamydial developmental cycle. By real-time reverse transcription polymerase chain reaction, the *pmpD* gene was found to be upregulated at 16 to 24 four hours after infection. Using polyclonal antibodies generated against the predicted passenger domain of PmpD, we demonstrated that it is initially localized on the surface of reticulate bodies, followed by its secretion outside *Chlamydia* starting at 24 hours after infection. In elementary bodies, we found a ≈157 kDa PmpD only inside the cell. Both events, the upregulation of *pmpD* gene transcription and PmpD protein processing and secretion, are coincidental with the period of replication and differentiation of RBs into EBs. We also demonstrated that, in the presence of penicillin, the cleavage and secretion of the putative passenger domain was suppressed.

**Conclusion/Significance:**

Our results are in agreement with the general concept that PmpD is an autotransporter protein which is post-translationally processed and secreted in the form of the putative passenger domain outside *Chlamydia* at mid- to- late point after infection, coinciding with the development of RBs into EBs.

## Introduction

*Chlamydia trachomatis*, an obligate intracellular parasite, is a major cause of bacterial sexually transmitted infections. *Chlamydia* has a unique developmental cycle with two distinct forms. The infectious form or elementary body (EB) is metabolically inactive. After entering the host cell, the EB develops into the noninfectious but metabolically active form called the reticulate body (RB). The proteins localized on the surface of these two different chlamydial particles are of particular interest because they are thought to play important roles in the interactions between *Chlamydia* and the host cell.

The genome of *C. trachomatis* serovar D described in 1998 [1] revealed much about this important human pathogen. One surprising finding was the discovery of a family of polymorphic membrane protein (*pmp*) genes in the small genome of *C. trachomatis*. Nine *pmp* genes have been found in *C. trachomatis* (serovar A/HAR13, D/UW-3) [1], [2] and *C. muridarum* (formerly *C. trachomatis* mouse pneumonitis) strain Nigg [3], twenty-one in *C. pneumoniae* strain CWL029 [4], seventeen in *C. pneumoniae* strain AR39 [3] and in *C. caviae* (formerly *C. psittaci* strain GPIC) [5], and eighteen in *C. abortus* S26/3 (formerly *C. psittaci* serovar 1) [6]. It was reported that the protein products of this family show similarity to other bacterial proteins which are either predicted or demonstrated to be autotransporters [7]. In general, chlamydial Pmps and autotransporter proteins share a signal sequence, a passenger domain containing amino acid motifs which define the function of the protein, and a carboxy beta-barrel (autotransporter domain). Autotransporter proteins are post-translationally processed beginning with the signal sequence which directs the protein from the cell cytoplasm across the inner membrane to the periplasm and is cleaved from the protein by signal peptidase I. The beta-barrel embeds in the outer membrane and facilitates the translocation of the passenger domain through the outer membrane. The passenger domain may be cleaved from the beta-barrel and is either bound to the bacterial membrane or secreted into the extracellular space [8], [9]. Similar to bacterial autotransporters, the chlamydial Pmps, which are predicted or described to be on the surface of *Chlamydia*, may play an important role in chlamydial infection and could be potential candidates in future vaccine designs [10]–[12]. Several publications recently demonstrated possible functions of Pmps in different *Chlamydia* species as mediating cell and humoral response to chlamydial infection [11], [13]–[18] and attachment and/or entry of EBs into an infected cell [18]–[21]. Much more work needs to be done to completely understand the nature, function, and localization of Pmps and their post-translational products in *Chlamydia.*

In this study we examined transcription of the *pmpD* gene, a member of the family of polymorphic membrane protein genes from *C. trachomatis* serovar L2, during the developmental cycle. We demonstrated by real-time reverse transcription polymerase chain reaction (RT-PCR) that the *pmpD* gene was upregulated at 16–24 hours after infection which coincides with the period of replication and differentiation of RBs into EBs. Using polyclonal antibodies generated against different PmpD fragments, we also demonstrated that the passenger domain of the *pmpD* gene product was initially localized on the surface of RBs but is no longer accessible to our antibodies when RBs convert into EBs. At the same time, the beta-barrel of PmpD was found embedded in the outer membrane of RBs and not fully accessible to our antibodies. In contrast to the surface localization of PmpD in RBs, we found the partially processed ≈157 kDa PmpD only inside EBs, probably in the periplasmic space. These findings shed some light on the important yet not fully understood role of PmpD in the developmental cycle of *C. trachomatis*.

## Methods

### Bacterial Strains

The *C. trachomatis* L2 (434/Bu) strain was used in these studies. McCoy cells (ATCC CRL 1696) were infected with *C. trachomatis* serovar L2, harvested at 24 and 48 hours (h) after infection, gently broken with a 2 ml Dounce Tissue Homogenizer (Bellco Biotechnology Inc., Vineland, NJ), and *Chlamydia* organisms were purified on a 30% Hypaque-76 (Renografin) (Nycomed Inc., Princeton, NJ) density gradient followed by purification through a 30–65% discontinuous Renografin gradient. The material collected after 48 h of infection was further treated with 0.25% Nonidet P-40 (Sigma Aldrich, St.Louis, MO) in phosphate buffer saline (PBS) to eradicate any remaining RBs [22]. The purity of each chlamydial population was verified with fluorescent-labeled monoclonal antibodies (MicroTrack^®^ *Chlamydia trachomatis* Direct Specimen Test, Trinity Biotech USA, Jamestown, NY) according to the manufacturer's instructions. The strain's serotype was confirmed with monoclonal antibodies in an inclusion typing method prior to use [23]. *E. coli* DH5α (Invitrogen, Gaithersburg, MD) was used for plasmid transformations and *E. coli* BL21 (DE3) (Novagen, Madison, WI) for overexpression of the recombinant fragments of *C. trachomatis* serovar L2 PmpD protein.

### Quantitation of *pmpD* gene expression by real time RT-PCR

McCoy cells were grown in 75 cm^2^ tissue culture flasks (CORNING Inc., Corning, NY) until full confluence and infected with *C. trachomatis* serovar L2 at a MOI of 0.33 with constant shaking at 37°C to ensure uniform infection. After infection, flasks were incubated in Eagle's minimal essential medium (Sigma Aldrich) supplemented with 1 µg/ml L-glutamine, 0.5 µg/ml cycloheximide, and 10% fetal bovine serum (HyClone, Logan, UT) at 37°C in a 5% CO_2_ environment. Total RNA was isolated from infected McCoy cells at 2, 4, 6, 8, 10, 12, 16, 20, 24, 30, 35, 48 hours post infection (p.i.) (one flask per each time point) using the RNeasy Mini Kit (QIAGEN, Valencia, CA) according to the manufacturer's instructions. RNA was also isolated from a monolayer of uninfected McCoy cells and from purified *C. trachomatis* serovar L2 EBs harvested at 48 h p.i. RNA was treated with RQ1 RNase-Free DNase (Promega, Madison,WI) for 30 min at 37°C, followed by phenol-chloroform extraction and ethanol precipitation. DNase treated RNA was tested by PCR to ensure complete DNA removal. Randomly primed cDNA synthesis was performed on 3–4 µg of RNA from each time point using SuperScript II RNase Reverse Transcriptase (Invitrogen Corp., Carlsbad, CA) and random hexamer primers (Promega), then purified using the QIAquick PCR Purification Kit (QIAGEN) according to the manufacturer's instructions. Chlamydial genomic DNA was isolated from purified EBs of *C. trachomatis* serovar L2 according to standard procedures [24]. Genomic DNA and cDNAs were quantified, aliquoted, and stored at −70°C prior to use. Real time RT-PCR was conducted in a LightCycler Instrument (Roche Applied Science, Indianapolis, IN) using the following primer sets (ct16s RNA-Forward (F):5′-GGAGAAAAGGGAATTTCACG; ct16s RNA-Reverse(R): 5′-TCCACATCAAGTATGCATCG (amplicon size 173bp)) [25] and *pmpD* (pmpD-F: 5′-TGTAGTTTTACGAGCAGCAACC; pmpD-R: 5′-AGATGATCATTCGCACTAGACC (amplicon size 320bp)) synthesized by QIAGEN Operon (Alameda, CA). The sequence of both *pmpD* gene primers was compared to the total preliminary genome of *C. trachomatis* serovar L2 available at www.sanger.ac.uk and the genome of *C. trachomatis* serovar D completed in 1998 [1] and obtained at www.stdgen.lanl.gov to ensure primer specificity. Briefly, 100ng of cDNA representing each time point or genomic DNA diluted 10-fold from 10^−1^ to 10^−7^ with known copy numbers based on the chlamydial genomic DNA molecular mass were amplified in a reaction mixture containing 0.5 µM of each gene specific primer, 3 mM MgCl_2_, and 2 µl of FastStart DNA Master SYBR Green I (Roche). Cycling parameters were as follows: 1 cycle at 95°C for 10 min and 45 cycles of 5 sec at 95°C, 10 sec at 65°C, 10 sec at 72°C, and 2 sec at 78°C during which fluorescence was measured. A melting curve for each amplicon was analyzed to ensure product specificity. The LightCycler 3 Run software (Version 4.24) generated a gene specific standard curve based on serial dilutions of the genomic DNA and copy numbers in all samples representing each time point were quantified. Uninfected McCoy cell cDNA and water as template were used as negative controls in each assay. A minimum of three PCR assays were performed for each time point using each gene specific primer set and the calculated copy numbers for each gene at each time point were then averaged. Relative *pmpD* expression at each time point was calculated as follows: the average *pmpD* copy number at each time point was divided by the average 16s RNA copy number at the corresponding time point and multiplied by 1×10^6^ [26]. Statistical error was calculated using the standard error of the mean [26].

### Cloning and expression of the recombinant fragments of PmpD and antibody production

**Figure 1:**
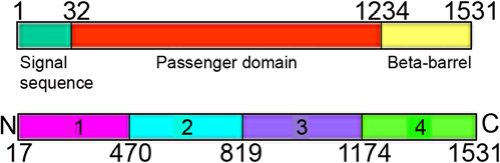
The fragments of PmpD of *C. trachomatis* serovar L2 used to raise antibodies and their correlation with the different domains of PmpD. Fragment 1, aa 17-517; fragment 2, aa 470-818; fragment 3, aa 819-1180; fragment 4, aa 1174-1531. Note: some fragments have overlapping aa sequence (1and 2; 3 and 4) and aa numbers show the beginning of each fragment.

The *pmpD* gene sequence was divided into four fragments encoding corresponding fragments of the PmpD protein. Fragments 1, 2, and 3 form the passenger domain of PmpD (the N-terminal portion) and fragment 4 the beta-barrel (the C-terminal portion) (Fig. 1). Primers used for amplification of each of these fragments of the *pmpD* gene are listed in Table 1. The conditions for PCR were set as follows: 1 cycle at 94°C for 2 min, 10 cycles of 15 sec at 94°C, 30 sec at 45°C, and 1 min at 72°C, and 10 cycles of 15 sec at 94°C, 30 sec at 45°C, and 1 min plus 20 sec/cycle at 72°C. Plasmid pML48 [27] was used as template DNA. Each amplified product was cloned into the pET17b vector (Novagen, Madison, WI) digested with *Nde*I and *Kpn*I. *E. coli* BL21 (DE3) was transformed and protein expression was performed according to the manufacturer's instructions. Production of each PmpD protein fragment was verified in a Western blot with anti-His-Tag monoclonal antibodies (CLONTECH Laboratories, Palo Alto, CA) and purified on a 1 ml HiTrap Chelating HP column (Amersham Biosciences AB, Uppsala, Sweden) according to the manufacturer's instructions. One mg of each purified protein was used for rabbit (fragment 1, 2, and 3) or goat (fragment 4) immunization (Kent Laboratories, Inc., Bellingham, WA). Immunoglobulins against each PmpD fragment were purified using 1 ml HiTrap Protein A HP column (Amersham Biosciences AB) and their reactivity was tested in a Western blot with each of the recombinant PmpD fragments, total lysates from *E. coli* transformed with the vector pET17b alone, and purified RBs and EBs of *C. trachomatis* serovar L2.

**Table 1:** Primers used for each *pmpD* gene fragment amplification.

PmpD fragm.	Primers
1	F: 5′-AAAAGCCATATGCATCACCATCACCATCACGTAGTAGCAGCTATCCTTGCC-3′;
	R: 5′-AGCTAAAATCGCTCCTCCTCCCAGAATTAAGGTACCCGAAGCAAAAGTCTTCAC-3′;
2	F: 5′-TCAGATCATATGCATCACCATCACCATCACCAAATGGAGTACAGGGAGGA-3′;
	R: 5′-GCCGCCATAAATATCAGAGAAGTTATTCGATAAGGTACCGGCCTCTTG-3′;
3	F: 5-TCGAATCATATGCATCACCATCACCATCACGCCATTTTTACAGGTTCTCTTC-3′;
	R: 5′-ATGGCCGTATGTGTCTTCTTCAATCTCTAAGGTACCTGCTACATGGAA-3′;
4	F: 5′-TTACAGCATATGCATCACCATCACCATCACGAAGAAGACACATACGGCCACATG-3′;
	R: 5′-GGGAGATTAGGGATAGAATGGTTGCTGTAAGGTACCCCAATC-3′;

### Immunofluorescence (IMF) microscopy

To determine PmpD localization in *C. trachomatis*, McCoy cells grown on coverslips were infected with *C. trachomatis* serovar L2. At 24 and 48 h p.i., cells were fixed with 100% methanol, washed in PBS, and reacted with antibodies against each fragment of PmpD, anti-MOMP polyclonal antibodies (pAb) (ViroStat, Portland, ME), and rabbit pre-immune sera diluted in 3% bovine serum albumin (BSA) in PBS for 1 h at room temperature. After washing in PBS, primary stained monolayers were reacted with corresponding fluorescent-conjugated secondary antibodies (Sigma-Aldrich, St. Louis, MO) diluted in 3% BSA-PBS. In addition, purified RBs and EBs of *C. trachomatis* were spotted on glass slides and stained directly with the antibodies mentioned above. Coverslips and slides were washed several times, mounted, and examined using a Leica epi-fluorescent microscope.

### Fractionation of McCoy cells infected with *C. trachomatis* serovar L2

McCoy cell monolayers in six-well tissue culture plates (CORNING Inc., Corning, NY) were inoculated to achieve 100% infectivity with *C. trachomatis* serovar L2 by centrifugation at 1,200×g at 37°C to ensure uniform infection. After centrifugation, the plates were incubated in Eagle's minimal essential medium as described above. At 6, 12, 24, 36, and 48 h p.i., the tissue culture medium was aspirated from each well, and the cells were overlaid with an equal amount of SPG containing protease inhibitors, dislodged with a cell scraper, and harvested. A Dounce Tissue Homogenizer was used to break the cells gently and protect the integrity of the *Chlamydia* particles. Each suspension was centrifuged at 125,000×g to separate the supernatant containing McCoy cell cytoplasmic proteins and secreted chlamydial proteins (soluble fraction) and the pellet containing the *Chlamydia* organisms and McCoy cell debris (insoluble fraction). Pellets were washed twice in PBS to remove the proteins present in the corresponding soluble fraction and resuspended in 100μl of Laemmli sample buffer [28] for SDS-PAGE. The proteins present in each soluble fraction were precipitated with TCA, washed twice in cold acetone, and resuspended in the same volume of Laemmli buffer.

### SDS-PAGE and Western blotting

The volume of each insoluble fraction loaded onto a 10% gel was pre-adjusted so that the amount of MOMP was equal in each preparation, ensuring that equal numbers of chlamydial organisms were examined [29]. Subsequently, each soluble fraction was loaded onto a gel using the same volume as the insoluble fraction prepared at the corresponding time point. Because the number of *Chlamydia* was still very low at 6 and 12 h p.i., the maximum loadable amounts of insoluble and soluble proteins prepared at these two time points were loaded on gels. Chlamydial proteins separated by SDS-PAGE were transferred to a nitrocellulose membrane, blocked, and washed according to standard procedures [24]. Each primary antibody, anti-MOMP pAb, or antibodies against fragment 2 of PmpD was diluted in 2.5% non-fat milk-Trizma buffer saline-Tween 20 (NFM-TBST). After multiple washes in TBST, the membrane was incubated with the corresponding secondary antibody conjugated with alkaline phosphatase (Sigma-Aldrich) diluted in 2.5% NFM-TBST, washed, and developed with BCIP/NBT (5-Bromo-4-Chloro3′-Indolylphosphate p-Toluidine salt/Nitro-blue Tetrazolium Chloride) according to the manufacturer's instructions (Pierce, Rockford, IL). Whenever required, the quantities of MOMP and PmpD in corresponding protein bands were determined and compared using the Packard Instrument OptiQuant software (version 03.10).

### Examination of penicillin action on PmpD processing

To examine the effect of penicillin on PmpD processing, McCoy cell monolayers in a six-well tissue culture plate (CORNING Inc.) were infected with *C. trachomatis* serovar L2 as described above. After infection, each well was overlaid with culture medium containing 0, 0.01, 0.1, 1.0, and 10.0 U/ml of penicillin (Sigma-Aldrich) respectively. The soluble and insoluble fractions of *Chlamydia* infected cells harvested at 24 h p.i. were prepared, and proteins in each fraction were loaded and separated by SDS-PAGE as described above. A nitrocellulose membrane with transferred proteins was reacted with anti-MOMP pAb or antibodies against fragment 2, followed by estimation of the quantities of MOMP and PmpD in the corresponding protein bands. In a separate experiment, the culture medium containing different concentrations of penicillin was replaced with medium without penicillin at 24 h p.i. and the cultures were incubated for 24 more hours. At 48 h p.i., soluble and insoluble fractions were prepared as described and analyzed by Western blot with antibodies against fragment 2 of PmpD.

## Results

### Determination of *pmpD* transcription by RT-PCR analysis

**Figure 2:**
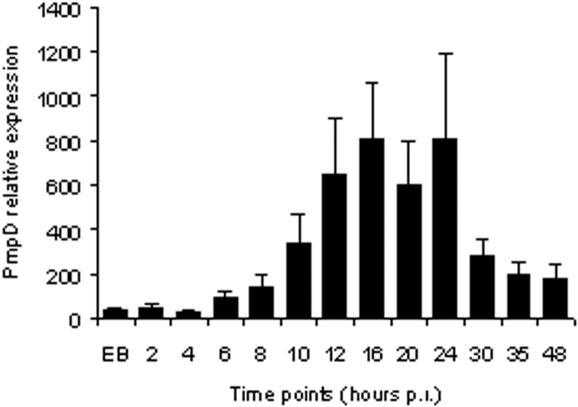
Relative transcription of *PmpD.* Total RNA was isolated from *C. trachomatis* serovar L2 infected McCoy cells at 0, 2, 4, 6, 8, 10, 12, 16, 20, 24, 30, 35, and 48 h p.i., random primed, and assayed by real time RT-PCR for 16S rRNA and *pmpD* copy number. Averaged *pmpD* copy numbers were indicated relative to the rRNA numbers at the same time points. Error bars were based on the standard error of the mean [26].

The kinetics of transcriptional expression of *pmpD* were studied using real-time PCR of cDNA synthesized from randomly primed total RNA isolated from *C. trachomatis* infected McCoy cells at 2, 4, 6, 8, 10, 12, 16, 20, 24, 30, 35, 48 h p.i. Because of the relatively constant expression of the 16S rRNA throughout the chlamydial developmental cycle, its level at each time point was used for normalization of the *pmpD* mRNA levels at the corresponding time points p.i. [25]. Very low levels of *pmpD* transcript were detected from 2 to 6 h p.i. (Fig. 2). This low level may be due to RNA found in EBs [30], since the same low level of *pmpD* expression was observed in purified chlamydial EBs. A significant increase in *pmpD* transcription was observed at 10 h p.i. during the logarithmic growth of chlamydial RBs and this expression increased through 16 h and leveled out from 16 to 24 h p.i. After 24 h p.i., *pmpD* expression decreased, but the final level was well above the very low level of *pmpD* expression observed at early time points and in purified chlamydial EBs, probably due to RBs of *C. trachomatis* that were still present.

### Characteristics of antibodies generated against recombinant protein fragments of PmpD

**Figure 3:**
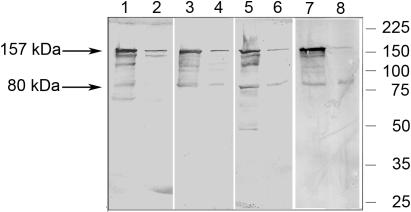
Reaction of pAb generated against PmpD recombinant protein fragments with total protein lysates prepared from purified RBs and EBs of *C. trachomatis* serovar L2. RBs (Lanes 1, 3, 5, and 7) and EBs (Lanes 2, 4, 6, and 8) were normalized against MOMP as described in METHODS. Lanes 1 and 2, pAb against fragment 1; Lanes 3 and 4, pAb against fragment 2; Lanes 5 and 6, pAb against fragment 3; Lanes 7 and 8, pAb against fragment 4. Protein markers in kDa are on the right side of the Western blot image.

To verify specificity of each of the four purified immunoglobulins generated against the recombinant protein fragments of PmpD, we tested them in a Western blot with each protein fragment and total proteins from *E. coli* transformed with the vector pET17b alone. The antibodies reacted only with the corresponding protein against which they had been generated and no reaction was observed with proteins from *E. coli* containing the vector pET17b (not shown). Each antibody was then reacted with total protein lysates prepared from purified RBs and EBs (Fig. 3) which were normalized so that the amount of MOMP was equal in each preparation [29]. We found that all four anti-PmpD antibodies reacted with the same ≈157 kDa protein present in a lysate prepared from RBs or EBs. To identify this ≈157 kDa protein, the band containing the protein of interest was excised from a Coomassie stained gel loaded with an aliquot of a total EB lysate of *C. trachomatis* serovar L2 and subjected to microspray column chromatography [31]. A non-redundant protein sequence database was searched directly with tandem mass spectra data using the computer program, SEQUEST [32]. The ≈157 kDa protein band contained peptides with identity to PmpD, indicating that this ≈157 kDa chlamydial protein is the product of the *pmpD* gene. It is important to note that mass spectrometry did not reveal any peptides derived from the signal sequence of the ≈157 kDa form of PmpD. The reaction with PmpD present in material prepared from RBs was much stronger with these antibodies than the reaction with the same protein in EBs, indicating its greater abundance in RBs than in EBs relative to MOMP. Smaller protein bands which reacted with all antibodies may represent post-translational products of PmpD, degradation products, or non-specific reaction of antibodies. Additional experiments are currently under way to identify these protein bands.

### Immunofluorescence microscopy

**Figure 4:**
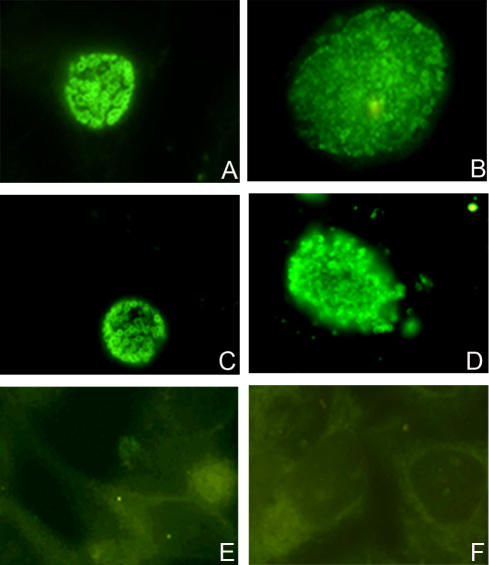
Fluorescent photographs of McCoy cells infected with *C. trachomatis* serovar L2. McCoy cells were methanol fixed at 24 (A, C, and E), and 48 (B, D, and F) h p.i. and probed with pAb against fragment 2 of PmpD (A and B), anti-MOMP PAb (C and D) and rabbit pre-immune sera (E and F). Ring shaped RBs of *Chlamydia* stained with anti-PmpD pAb indicate surface localization of PmpD in *Chlamydia*. Note: the lack of staining in the infected cell cytoplasm indicating that PmpD is not transported outside the inclusion. The Figure was scanned using Image Pro-Plus 4.1 and prepared with Adobe Photoshop 6.0.

**Figure 5:**
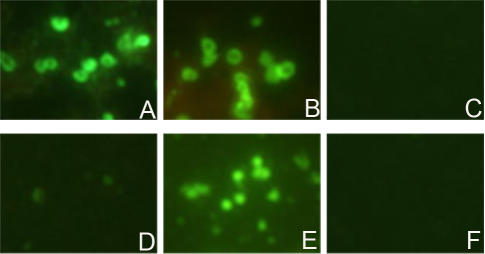
Fluorescent photographs of unfixed RBs and EBs of *C. trachomatis* serovar L2. RBs (A, B, and C) and EBs (D, E, and F) were harvested and purified at 24 and 48 h p.i. respectively, and probed with pAb against fragment 2 of PmpD (A and D), anti-MOMP pAb (B and E), and rabbit pre-immune sera (C and F). Staining of EBs with anti-PmpD pAb was negligible while anti-MOMP Pab stained both RBs and EBs indicating that the PmpD passenger domain may be secreted during the conversion of RBs into EBs. The Figure was scanned using Image Pro-Plus 4.1 and prepared with Adobe Photoshop 6.0.

To examine the cellular localization of PmpD, McCoy cells infected with *C. trachomatis* serovar L2 and fixed with methanol at 24 and 48 h p.i. were reacted with antibodies against fragment 2 of PmpD (Fig. 4). Very bright doughnut-shaped fluorescing chlamydial particles, representing RBs, were observed in McCoy cell monolayers at 24 h p.i. An identical pattern was observed with antibodies against fragment 1, 3, and 4 (not shown). To determine if a portion of PmpD was secreted into the host cell cytoplasm, we followed the fixation procedure described by Vandahl et al [33]. Fixed cell monolayers were reacted with all antibodies mentioned above, but no staining was observed outside the inclusion membrane (not shown). In contrast to the very sharp staining of *Chlamydia* inside each inclusion at 24 h p.i. with anti-PmpD antibodies, staining of inclusions at 48 h p.i. was diffuse and it was difficult to distinguish individual chlamydial particles at this time point. To resolve this issue, purified chlamydial RBs and EBs were reacted with antibodies (Fig. 5). Methanol fixed and unfixed EBs did not stain when reacted with antibodies against all four fragments of PmpD but reacted with anti-MOMP PAbs. Contrary to EBs, both fixed and unfixed RBs reacted with antibodies against fragment 1, 2, and 3 of PmpD and MOMP which was used as a control for the surface localization of a chlamydial protein [34]. At the same time, antibodies against fragment 4 of PmpD reacted with RBs only after methanol fixation (not shown).

### PmpD translation, processing, and secretion

**Figure 6:**
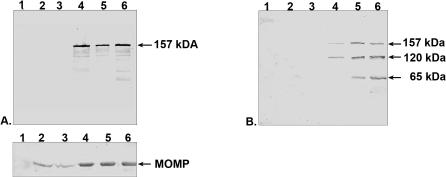
PmpD translation, processing, and secretion. McCoy cells infected with *C. trachomatis* serovar L2 were harvested at 6, 12, 24, 36, and 48 hours after infection and soluble and insoluble protein fractions separated. A. The insoluble fractions were loaded for equal MOMP amounts. B. The soluble fractions were loaded as described in METHODS. Both nitrocellulose membranes were reacted with antibodies against fragment 2 of PmpD. Lane 1, 0 h p.i. (uninfected cells). Lane 2, 6 h p.i. Lane 3, 12 h p.i. Lane 4, 24 h p.i. Lane 5, 36 h p.i. Lane 6, 48 h p.i. Reaction of MOMP used for the total protein normalization in the insoluble fractions, with anti-MOMP pAb is shown in a separate box. The quantity of MOMP was determined using the Packard Instrument OptiQuant software (version 03.10).

Total proteins from *C. trachomatis* infected cells were harvested at 6, 12, 24, 36, and 48 h p.i. and separated into soluble and insoluble fractions by high speed centrifugation. After being normalized against MOMP so that equal numbers of chlamydial organisms were examined, the total proteins in the insoluble fractions were transferred to a nitrocellulose membrane and incubated with antibodies against fragment 2 of PmpD (Fig. 6A). A very strong reaction was observed with the ≈157 kDa PmpD protein band present in the insoluble fractions prepared at 24, 36, and 48 h after infection.

**Figure 7:**
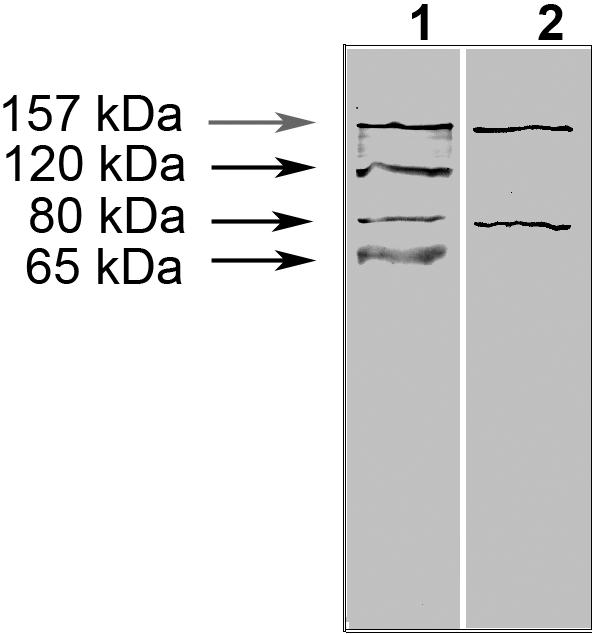
Reaction of pAb generated against the C- and N- terminal portions of PmpD with the soluble protein fraction purified at 48h p.i. The soluble fraction was prepared as described in METHODS. Lane 1, pAb against fragment 2 (part of the passenger domain); Lane 2, pAb against fragment 4 (the beta-barrel).

**Figure 8:**
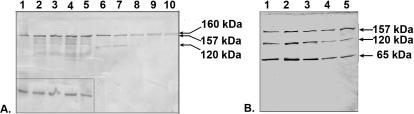
Processing of PmpD is inhibited in the presence of penicillin (A) and restored upon its removal (B). McCoy cells infected with *C. trachomatis* serovar L2 and incubated in the culture medium containing different doses of penicillin were harvested at 24 (A) or 48 (B) h p.i. and soluble and insoluble protein fractions separated. Both nitrocellulose membranes were reacted with pAb against fragment 2 of PmpD. A. Lines 1–5, the insoluble fractions were loaded for equal MOMP amounts (reaction with MOMP is shown in the small box). Lines 6–10, soluble fractions were loaded as described in METHODS. Lane 1 and 6, 0 penicillin. Lane 2 and 7, 0.01 U/ml penicillin. Lane 3 and 8, 0.1 U/ml penicillin. Lane 4 and 9, 1 U/ml penicillin. Lane 5 and 10, 10 U/ml penicillin. Note: a doublet of protein bands with molecular weights of ≈160 and 157 kDa is visible in both the insoluble and soluble fractions corresponding to penicillin concentrations of 0.1 U/ml and higher. B. Soluble fractions were loaded as described in METHODS. Lane1, 0 penicillin. Lane 2, 0.01 U/ml penicillin. Lane 3, 0.1 U/ml penicillin. Lane 4, 1 U/ml penicillin. Lane 5, 10 U/ml penicillin (indicated concentrations of penicillin were used before penicillin removal at 24 h p.i.). The quantity of MOMP and PmpD was determined using the Packard Instrument OptiQuant software (version 03.10).

When the total proteins in each soluble fraction were reacted with antibodies against fragment 2 of PmpD, ≈157, 120, and 65 kDa protein bands were observed in the 24, 36 and 48 h fractions (Fig. 6B). The ≈120 kDa protein reacted weakly in the 24 h p.i. soluble fraction, while a much stronger reaction was found at the later time points after infection. A similar reaction pattern was observed with the ≈65 kDa protein band starting at 36 h p.i. The ≈157 kDa protein found in both the soluble and insoluble fractions probably results from leakage of proteins due to damage to some of the *Chlamydia* organisms that occurred during the fractionation of McCoy cells infected with *C. trachomatis*, since the chlamydial cytoplasmic HSP60 protein was also found in the soluble fractions with antibodies against this protein (kindly provided by Richard Morrison) (not shown). In contrast, it is important to note that the ≈120 and 65 kDa proteins were found exclusively in the soluble fractions indicating their extra-cellular localization. Antibodies against fragments 1 and 3 reacted similarly to antibodies against fragment 2 (not shown). However, when total proteins in the soluble fraction prepared at 48 h p.i. were reacted with antibodies against fragment 4 (the beta-barrel), the ≈120 and 65 kDa proteins did not react. (Fig. 7). No signal was observed with uninfected McCoy cells used as a negative control. When soluble and insoluble fractions prepared from *Chlamydia* infected cells incubated with different concentrations of penicillin in the culture medium and harvested at 24 h p.i. were reacted with antibodies to fragment 2 of PmpD, the ≈120 kDa protein band disappeared at penicillin concentrations of 0.1U/ml and higher (Fig. 8A). At the same time, a doublet of protein bands with molecular weights of ≈160 and 157 kDa, appeared in the insoluble fractions corresponding to penicillin concentrations of 0.1 U/ml and higher, leading to a two-fold increase of the combined 160–157 kDa proteins compared with the fraction without penicillin. When we analyzed the soluble fractions prepared from McCoy cells infected with *Chlamydia* and incubated in the presence of different concentrations of penicillin during the first 24 h and in the absence of penicillin for the second 24 h, we found that secretion of the ≈120 kDa protein was restored upon removal of penicillin (Fig. 8B).

## Discussion

In this work, we studied *pmpD* gene expression and protein location in *C. trachomatis* serovar L2 during its developmental cycle. When we examined the relative transcription of the *pmpD* gene, we found its maximal expression occurred in the mid-period of the chlamydial developmental cycle which coincides with the growth and differentiation of RBs. The reduced level of *pmpD* expression at late and very late time points after infection (30–48 hours) could be explained by the conversion of most RBs to infectious EBs, in which little *pmpD* expression occurs. The pattern of transcription that we detected by real-time RT-PCR was in agreement with the results of other investigators who used different approaches to study *pmp* D gene transcriptional profiling during the developmental cycle of *C. trachomatis* serovar D and L2 and *C. pneumoniae* strain CWL029 [35]–[37]. Interestingly, a very similar pattern of the relative expression of another *pmp* gene in *C. trachomatis*, the *pmpC* gene, was described earlier [38].

When PmpD localization during the chlamydial developmental cycle was studied with IMF microscopy using antibodies against different fragments of PmpD, we found the N-terminal portion of PmpD (the passenger domain) on the surface of RBs and the C-terminal portion (the beta-barrel) possibly embedded in the outer membrane of RBs and accessible to antibodies only after the membrane was permeabilized with methanol. This pattern of differential staining of chlamydial RBs with antibodies generated against the N- and C-terminal portions of PmpD agrees with an autotransporter secretion mechanism of PmpD. At the same time, these antibodies failed to reveal the presence of PmpD in EBs in IMF microscopy. Based on the autotransporter protein secretion model [9], [39] and the absence of peptides comprising the signal sequence of PmpD, we believe that the ≈157 kDa protein found in a total lysate of purified EBs (Fig 3) represents the periplasmic form of PmpD that was not further processed before reorganization of RBs into EBs when structural changes in the chlamydial outer membrane take place [40]. Disulfide bonds cross-link the proteins localized in the periplasm and outer membrane and would prevent an autotransporter protein from folding correctly and translocating subsequently across the outer membrane [41], [42]. The quantity of the ≈157 kDa PmpD protein remaining in the periplasm is probably very low since no measurable staining of individual EBs was observed in IMF microscopy with our antibodies even when EBs were fixed with methanol. However, when proteins from a large number of EBs were loaded onto a gel, the ≈157 kDa protein was detected by immunoblot. A similar detection pattern was observed for a predicted autotransporter protein in RBs and EBs, the Cpn0796 protein from *C. pneumoniae*, which was found on the surface of organisms in young inclusions but not on the surface of purified EBs harvested at late time points after infection while the protein without the signal sequence, was found in immunoblots of total EB lysates [33]. PmpD with a molecular weight of 167 kDa (the full length protein including the signal sequence has a molecular weight of 170.8 kDa) was also found in immunoblot of EBs of *C. pneumoniae* [43], thus corroborating our finding of partially processed PmpD in chlamydial EBs.

The ≈120 kDa protein found in a Western blot from soluble fractions of *Chlamydia* infected McCoy cells prepared at 24, 36, and 48 h p.i. by antibodies against the N-terminal portion of PmpD matches the size of the predicted passenger domain of PmpD after the beta-barrel has been removed. Importantly, the ≈120 kDa protein did not react with antibodies generated against the beta-barrel of PmpD, supporting our idea that the ≈120 kDa protein represents the passenger domain of PmpD cleaved from the beta-barrel and secreted outside of *Chlamydia*. In additional support of our results, mass spectrometry analysis of the ≈120 and 65 kDa proteins found in the soluble fraction of *Chlamydia* infected cells revealed that these two proteins lacked the peptides forming the signal sequence and the beta-barrel of PmpD. In addition, the ≈65 kDa protein was a truncated version of the ≈120 kDa protein indicating further processing of the passenger domain after cleavage from the beta-barrel (manuscript in preparation). Our results are in accordance with the results of Wehrl et al [18] who reported that two proteins with molecular weight of 120–130 and 70 kDa, found in a total lysate of HEp-2 cells infected with *C. pneumoniae* prepared at mid- to- late time points after infection, were the posttranslational products of PmpD correlating to its N-terminus.

Cleavage of an autotransporter protein's signal peptide by signal peptidase I after the protein is translocated across the inner cytoplasmic membrane is an essential step for further processing [44]. Kuo et al [45] first reported that this enzyme could be effectively inhibited in *E.coli* by beta-lactam compounds. To determine whether cleavage of the signal peptide of PmpD could be blocked by penicillin, a beta-lactam antibiotic, leading to further inhibition of PmpD post-translational processing, we incubated *Chlamydia* infected cells in the presence of different doses of penicillin. We found the ≈160 kDa full length PmpD along with the partially processed ≈157 kDa protein in the corresponding insoluble fractions, indicating that cleavage of the signal peptide of PmpD was not fully suppressed by penicillin at the concentrations used in our study. We also found that the ≈120 kDa band, correlating to the predicted passenger domain of PmpD, disappeared in the soluble fractions at penicillin concentrations of 0.1 U/ml and higher indicating suppression of further post-translational processing of PmpD by penicillin. Blockage of the ≈120 kDa protein processing by penicillin indicates that this antibiotic affects other mechanisms involved in the post-translational processing of PmpD than signal peptide cleavage. The machinery of cleavage of the passenger domain remains controversial and has been described for only a few autotransporters to date [8], [46]. Several investigators demonstrated earlier that penicillin blocks conversion of RBs into EBs [47], [48]. It is possible that beta-lactams may affect PmpD processing and secretion either by inhibition of signal peptidase I, inhibition of the passenger domain processing, and/or blockage of the conversion of RBs into EBs. More experiments are currently under way to examine these possibilities. After penicillin withdrawal, the processing of the PmpD protein was restored. Similar results were obtained with another beta-lactam antibiotic, cefotaxime (not shown).

Recently PmpD was described as localized on the surface of EBs of *C. trachomatis* and functioning as a *C. trachomatis* species-common antigen [12]. Contrary to these results of Crane at al with EB staining, our antibodies generated against PmpD fragments failed to recognize EBs. At the same time, the rabbit anti-155 kDa antiserum used by Crane et al did not recognize the ≈157 kDa PmpD protein in a total EB lysate in a Western blot but reacted with smaller ≈80 and 42 kDa proteins [12]. We believe that the discrepancy in antibody reactivity could be due to the different structures of the PmpD proteins that were used to raise the antibodies, and, as a result, recognition of different epitopes in *Chlamydia* by the antibodies generated. We used linear protein fragments of PmpD produced in recombinant *E.coli* whereas Crane et al [12] used the entire native PmpD protein present in a Triton X-100 soluble fraction of *Chlamydia* harvested at 24 h after infection and purified by two-dimensional electrophoresis.

Based on the results of our investigations, we conclude that the *pmpD* gene is upregulated at 16–24 h p.i. and the PmpD protein is post-translationally processed and secreted in the form of the putative passenger domain outside *Chlamydia* at mid- to- late point after infection, coinciding with the development of RBs into EBs.

## References

[1] Stephens RS, Kalman S, Lammel C, Fan J, Marathe R (1998). Genome Sequence of an Obligate Intracellular Pathogen of Humans: *Chlamydia trachomatis*.. Science.

[2] Carlson JH, Porcella SF, McClarty G, Caldwell HD (2005). Comparative Genomic Analysis of *Chlamydia trachomatis* Oculotropic and Genitotropic Strains.. Infect Immun..

[3] Read TD, Brunham RC, Shen C, Gill SR, Heidelberg JF (2000). Genome sequences of *Chlamydia trachomatis MoPn* and *Chlamydia pneumoniae AR39*.. Nuc Acids Res..

[4] Kalman S, Mitchell W, Marathe R, Lammel C, Fan J (1999). Comparative genomes of *Chlamydia pneumoniae* and *Chlamydia trachomatis*.. Nature Genetics.

[5] Read TD, Myers GS, Brunham RC, Nelson WC, Paulsen IT (2003). Genome sequence of *Chlamydophila caviae* (*Chlamydia psittaci GPIC*): examining the role of niche-specific genes in the evolution of the *Chlamydiaceae*.. Nucleic Acids Res.,.

[6] Thomson NR, Corin Y, Bell K, Holden MT, Bentley SD (2005). The *Chlamydophila abortus* genome sequence reveals an array of variable proteins that contribute to interspecies variation.. Genome Res.

[7] Henderson IR, Lam AC (2001). Polymorphic proteins of *Chlamydia* spp. –autotransporters beyond the Proteobacteria.. Trends Microbiol..

[8] Henderson IR, Navarro-Garcia F, Desvaux M, Fernandez RC, Ala'Adeen D (2004). Type V Protein Secretion Pathway: the Autotransporter Story.. Microbiol Mol Biol Revews.

[9] Jose J (2006). Autodisplay: efficient bacterial surface display of recombinant proteins.. Appl Microbiol Biotechnol.

[10] Christiansen G, Pederson AS, Hjerno K, Vandahl BB, Birkelund S (2000). Potential Revelance of *Chlamydia pneumoniae* Surface Proteins to an Effective Vaccine.. J Inf Dis.

[11] Longbottom D, Russel M, Dunbar SM, Jones GE, Herring AJ (1998). Molecular Cloning and Characterization of the Genes Coding for the Highly Immunogenic Cluster of 90-Kilodalton Envelope Proteins from the *Chlamydia psittaci* Subtype That Causes Abortion in Sheep.. Inf Immun.

[12] Crane DD, Carlson JH, Fisher ER, Bavoil P, Ru-ching Hsia (2006). *Chlamydia trachomatis* polymorphic membrane protein D is a species-common pan-neutralizing antigen.. PNAS..

[13] Fling SP, Sutherland RA, Steele LN, Hess B, D'Orazio SE (2001). CD8+ T cells recognize an inclusion membrane-associated protein from the vacuolar pathogen *Chlamydia trachomatis*.. Proc Natl Acad Sci U S A..

[14] Goodall JC, Yeo G, Huang M, Raggiaschi R, Gaston JSH (2001). Identification of *Chlamydia trachomatis* antigens recognized by human CD4+ T lymphocytes by screening an expression library.. Eur J Immunol..

[15] Mygind T, Vandahl B, Pedersen AS, Christiansen G, Hollsberg P (2004). Identification of an in vivo CD4+ T-cell mediated response to polymorphic membrane proteins of *Chlamydia pneumoniae* during experimental infection.. FEMS Immunol Med Microbiol.

[16] Niessner A, Kaun C, Zorn G, Speidl W, Turel Z (2003). Polymorphic Membrane Protein (PMP) 20 and PMP21 of *Chlamydia pneumoniae* Induce Proinflammatory Mediators in Human Endothelial Cells In Vitro by Activation of the Nuclear Factor-kB Pathway.. J Infect Dis..

[17] Pedersen AS, Christiansen G, Birkelund S (2001). Differential expression of Pmp10 in cell culture infection with *Chlamydia pneumoniae* CWL029.. FEMS Microbiol Lett.

[18] Wehrl W, Brinkmann V, Jungblut PR, Meyer TF, Szczepek AJ (2004). From the Inside Out–Processing of the Chlamydial Autotransporter PmpD and Its Role in Bacterial Adhesion and Activation of Human Host Cells.. Mol Microbiol.

[19] Grimwood J, Stephens RS (1999). Computational Analysis of the Polymorphic Membrane Protein Superfamily of *Chlamydia trachomatis* and *Chlamydia pneumoniae*.. Micro Comparat Genom.

[20] Vandahl BB, Pedersen AS, Gevaert K, Holm A, Vandekerckhove J (2002). The expression, processing, and localization of polymorphic membrane proteins in *Chlamydia pneumoniae* strain CWL029.. BMC Microbiol,.

[21] Vretou E, Giannikopoulo P, Psarrou E (2001). Polymorphic outer-membrane proteins of *Chlamydophila abortus* are glycosylated.. Microbiol.

[22] Tanzer RJ, Longbottom D, Hatch TP (2001). Identification of Polymorphic Outer Membrane Proteins of *Chlamydia psittaci 6BC*.. Infect Immun.

[23] Suchland RJ, Stamm WE (1991). Simplified microtiter cell culture method for rapid immunotyping of *Chlamydia trachomatis*.. J Clin Microbiol.

[24] Maniatis T, Fritsch EF, Sambrook J (1989). Molecular cloning, a laboratory manual..

[25] Mathews SA, Volp KM, Timms P (1999). Development of a quantitative gene expression assay for *Chlamydia trachomatis* identified temporal expression of sigma factors.. FEBS Lett.

[26] Hogan RJ, Mathews SA, Kutlin A, Hammerschlag MR, Timms P (2003). Differential expression of genes encoding membrane proteins between acute and continuous *Chlamydia pneumoniae* infections.. Microbial Pathogen..

[27] Lampe MF, Ballweber LM, Stamm WE (1998). Cloning and sequence analysis of a putative cytolysin from *Chlamydia trachomatis*.. the Proceedings of the Ninth International Symposium on Human Chlamydial infection.

[28] Laemmly UK (1970). Cleavage of structural proteins during the assembly of the head of bacteriophage T4.. Nature.

[29] Tanzer RJ, Hatch TP (2001). Characterization of Outer Membrane Proteins in *Chlamydia trachomatis LGV* Serovar L2.. J Bacteriol.

[30] Douglas AL, Hatch TP (2000). Expression of the transcripts of the sigma factors and putative sigma factor regulators of *Chlamydia trachomatis* L2.. Gene..

[31] Gatlin CL, Kleemann GR, Hays LG, Link AJ, Yates JR (1998). Protein identification at the low femtomole level from silver-stained gels using a new fritless electrospray interface for liquid chromatography-microspray and nanospray mass..

[32] Eng JK, McCormack AL, Yates JR (1994). An approach to correlate tandem mass spectral data of peptides with amino acid sequences in a protein database.. J Am Soc Mass Spectrom.

[33] Vandahl BB, Stensballe A, Roepstorff P, Christiansen G, Birkelund S (2005). Secretion of Cpn0796 from *Chlamydia pneumoniae* into the host cell cytoplasm by an autotransporter mechanism.. Cell Microbiol.

[34] Birkelund S, Lundemose AG, Christiansen GA (1989). Characterization of Native and Recombinant 75-Kilodalton Immunogens from *Chlamydia trachomatis* Serovar L2.. Infect Immun..

[35] Belland RJ, Zhong G, Crane DD, Hogan D, Sturdevant D (2003). Genomic transcriptional profiling of the developmental cycle of *Chlamydia trachomatis*.. PNAS.

[36] Lindquist EA, Stephens RS (1998). Transcriptional activity of a sequence variable protein family in *Chlamydia trachomatis*.. Proc. of the Ninth International Symposium on Human Chlamydial Infection.

[37] Nicholson TL, Olinger L, Chong K, Schoolnik G, Stephens RS (2003). Global Stage Specific Gene Regulation during the Developmental Cycle of *Chlamydia trachomatis*.. J Bacteriol.

[38] Gomez JP, Ru-ching Hsia, Mead S, Borrego MJ, Dean D (2005). Immunorteactivity and differential developmental expression of known and putative *Chlamydia trachomatis* membrane proteins for biologically variant serovars representing distinct disease groups.. Microbes&Inf..

[39] Desvaux M, Parham NJ, Henderson IR (2004). The autotransporter secretion system.. Res in Microbiol.

[40] Moulder WJ (1991). Interaction of *Chlamydiae* and Host Cells in Vitro.. Microbiol Review,.

[41] Eppens EF, Nouwen N, Tommassen J (1997). Folding of a bacterial outer membrane protein during passage through the periplasm.. EMBO.

[42] Jose J, Zangen D (2005). Autodisplay of the protease inhibitor aprotinin in *Escherichia coli*.. Biochem Biophis Res Communic.

[43] Montigiani S, Falugi F, Scarselli M, Finco O, Petracca R (2002). Genomic Approach for Analysis of Surface Proteins in *Chlamydia pneumoniae*.. Infect Immun.

[44] Dalbey RE, Wickner W (1985). Leader Peptidase Catalyzes the Release of Exported Proteins from the Outer Surface of the *Escherichia coli* Plasma Membrane.. J. Bio Chem..

[45] Kuo D, Weidner J, Griffin P, Shah SK, Knight WB (1994). Determination of the kinetic parameters of *Escherichia coli* leader peptidase activity using a continuous assay: the pH dependence and time-dependent inhibition by beta-lactams are consistent with a novel serine protease mechanism.. Biochem.

[46] Dautin N, Barnard TJ, Anderson DE, Bernstein HD (2007). Cleavage of a bacterial autotransporter by an evolutionary convergent autocatalytic mechanism.. EMBO.

[47] Lee CK, Bowie WR, Alexander ER (1978). In Vitro Assays of the Efficacy of Antimicrobial Agents in Controlling *Chlamydis trachomatis* Propagation.. Antimicrob Agents Chemother..

[48] Barbour AG, Amano Ken-ichi, Hackstadt T, Perry L, Caldwell HD (1982). *Chlamydia trachomatis* Has Penicillin-Binding Protein but Not Detectable Muramic Acid.. J. Bacteriol..

